# Sapien ‘valve-in-valve’ implantation and valve leaflet resection for treating endocarditis of a bioprosthetic mitral valve: a case report

**DOI:** 10.1093/ehjcr/ytae078

**Published:** 2024-02-12

**Authors:** Aljona Friedrich, Miralem Pasic, Volkmar Falk

**Affiliations:** Deutsches Herzzentrum der Charité, Department of Cardiothoracic and Vascular Surgery, Augustenburger Platz 1, 13353 Berlin, Germany; Charité-Universitätsmedizin Berlin, Corporate Member of Freie Universität Berlin, Humboldt-Universität zu Berlin, Charitéplatz 1, 10117 Berlin, Germany; Deutsches Herzzentrum der Charité, Department of Cardiothoracic and Vascular Surgery, Augustenburger Platz 1, 13353 Berlin, Germany; Charité-Universitätsmedizin Berlin, Corporate Member of Freie Universität Berlin, Humboldt-Universität zu Berlin, Charitéplatz 1, 10117 Berlin, Germany; DZHK (German Center for Cardiovascular Research), Partner Site Berlin, Berlin, Germany; Deutsches Herzzentrum der Charité, Department of Cardiothoracic and Vascular Surgery, Augustenburger Platz 1, 13353 Berlin, Germany; Charité-Universitätsmedizin Berlin, Corporate Member of Freie Universität Berlin, Humboldt-Universität zu Berlin, Charitéplatz 1, 10117 Berlin, Germany; DZHK (German Center for Cardiovascular Research), Partner Site Berlin, Berlin, Germany; Department of Health Sciences and Technology, Institute of Translational Medicine, Translational Cardiovascular Technologies, Swiss Federal Institute of Technology (ETH) Zurich, Zurich, Switzerland

**Figure:**
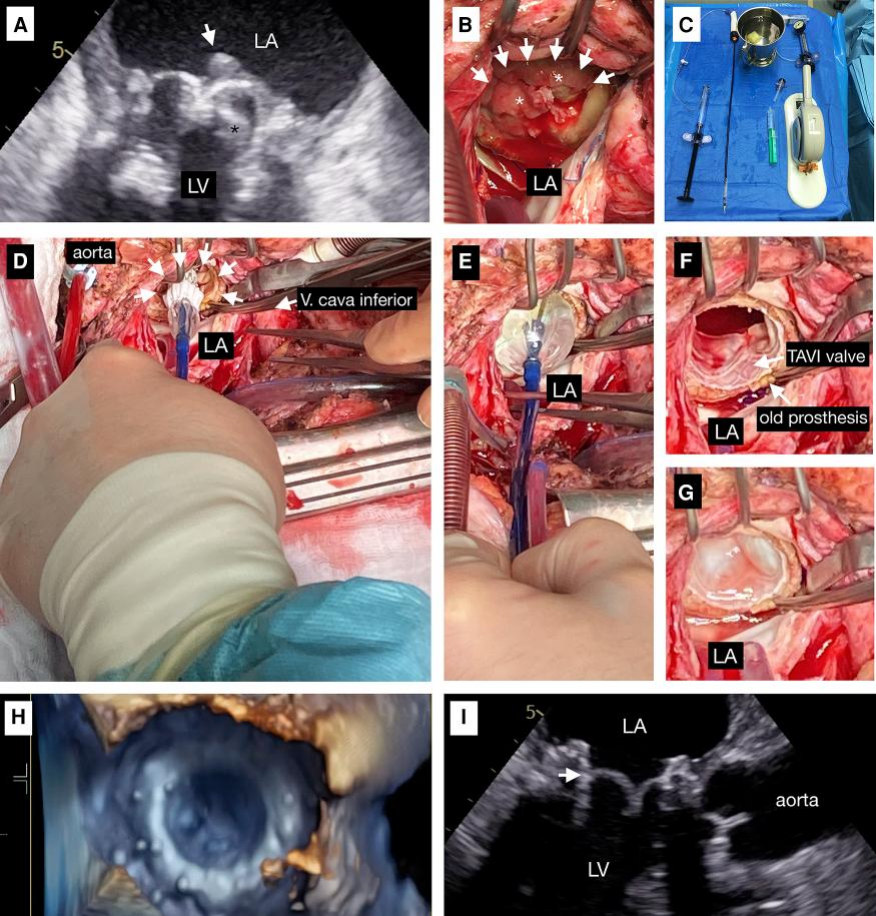


A 70-year-old female presented with fever, malaise, and dyspnoea at rest in November 2021. Her medical history included mitral valve replacement (*Mosaic size* 29 mm) in July 2019 due to mitral valve endocarditis (caused by *Staphylococcus hominis* and *Staphylococcus haemolyticus*) and redo bioprosthetic mitral valve (*Biointegral size* 33 mm) replacement in November 2020 due to chronic atrioventricular disconnection. In July 2021, she was diagnosed with breast cancer with abscess formation caused by *Enterococcus faecalis* that required surgical treatments.

On admission, echocardiography showed large floating vegetations originating from the leaflets of the bioprosthetic mitral valve (*Panel A*) but no recurrent atrioventricular dehiscence. Blood cultures revealed *E. faecalis*, the same bacteria as in the breast abscess.

During the third mitral valve surgery, vegetations were only seen at the leaflets of the bioprosthetic valve but not on the pericardial prosthetic ring (*Panel B*). Explantation of the bioprosthesis was considered too high risk, given the history of previous atrioventricular disconnection. Therefore, the surgical strategy was modified and only the leaflets of the mitral bioprosthesis were excised, and a 29* *mm Sapien valve was implanted as ‘valve in valve’ inside the old mitral bioprosthesis under direct vision using a standard transapical delivery system (*Panel C*). The valve was implanted inside the remaining mitral valve prosthetic ring (*Panels D* and *E*). *Panel F* shows the implanted new valve (*TAVI-Valve Sapien* 3 29 mm) in the old mitral valve prosthesis (*Biointegral* 33 mm). The leaflets of the old prosthesis are excised. The new valve (TAVI Sapien 3) is open, and the leaflets are not visible. *Panel G* shows the new valve closed, and the three leaflets are visible.

After additional antibiotic treatment, the patient recovered well and returned to everyday life. The patient received piperacillin–tazobactam and ceftriaxone preoperative for 10 days and postoperative meropenem, vancomycin, and rifampicin for 2 weeks such as gentamycin, ampicillin, and ceftriaxone for 5 days and ampicillin, ceftriaxone, and fosfomycin for 10 days. Afterwards the patient received ampicillin and ceftriaxone for another 2 weeks plus additional amoxicillin and doxycycline for 1 year after surgery.

A follow-up echocardiography showed no signs of endocarditis and a good function of the Sapien valve (*Panels H* and *I*). Two years after the procedure, she is free from fever and other signs of inflammation.

Sapien ‘valve-in-valve’ implantation and leaflet resection of a bioprosthesis was used as bailout procedure for treatment of endocarditis after bioprosthetic mitral valve replacement in order to reduce very high surgical risk.

## Supplementary Material

ytae078_Supplementary_Data

## Data Availability

The data underlying this article are available in the article and in its online Supplementary material.

## Supplementary material

**Consent:** The authors confirm that informed written consent was obtained for the submission and publication of this case report, including the accompanying text and images, as per COPE guidelines.

**Funding:** This research received no specific grant from any funding agency in the public, commercial, or not-for-profit sectors.

