# Supplementary material for: Sapien ‘valve-in-valve’ implantation and valve leaflet resection for treating endocarditis of a bioprosthetic mitral valve: a case report
Source: Eur Heart J Case Rep. 2024 Feb 12;8(2):ytae078. doi: 10.1093/ehjcr/ytae078 (PMC10894001; doi:10.1093/ehjcr/ytae078)
Supplement: ytae078_Supplementary_Data [file ytae078_supplementary_data.zip › abstract_friedrich_pasic_falk.docx]

**Sapien “valve-in-valve” implantation and valve leaflet resection for treating endocarditis of a bioprosthetic mitral valve – case report**

Abstract:

A 70-year-old female needed third-time redo mitral valve replacement due to endocarditis. As she was considered too high risk for conventional replacement, she underwent implantation of a Sapien 3 transcatheter valve prosthesis as Valve in Valve under direct vision.

Background:

Endocarditis of a prosthetic valve (PVI) is a very serious condition with often poor prognosis and a high in-hospital mortality. Especially patients with multiple co-morbidities including older age or intracardiac abscess are at risk. As therapeutic strategies are still debated and there are no evidence-based recommendations, surgery is recognized as the best option for patents with PVI. Mostly including removal of all foreign material to prevent relapse (1). This special case is presenting a reinfected endocarditis with limited options of surgical strategy and a therapeutic approach which hadn’t been reported in the literature so far.

REFERENCES

1. Victoria Delgado, Nina Ajmone Marsan, Suzanne de Waha, Nikolaos Bonaros, Margarita Brida, Haran Burri, Stefano Caselli, Torsten Doenst, Stephane Ederhy, Paola Anna Erba, Dan Foldager, Emil L Fosbøl, Jan Kovac, Carlos A Mestres, Owen I Miller, Jose M Miro, Michal Pazdernik, Maria Nazarena Pizzi, Eduard Quintana, Trine Bernholdt Rasmussen, Arsen D Ristić, Josep Rodés-Cabau, Alessandro Sionis, Liesl Joanna Zühlke, Michael A Borger, ESC Scientific Document Group , 2023 ESC Guidelines for the management of endocarditis: Developed by the task force on the management of endocarditis of the European Society of Cardiology (ESC) Endorsed by the European Association for Cardio-Thoracic Surgery (EACTS) and the European Association of Nuclear Medicine (EANM), European Heart Journal, Volume 44, Issue 39, 14 October 2023, Pages 3948–4042, <https://doi.org/10.1093/eurheartj/ehad193>

Key words: case report, mitral valve endocarditis, recurrent endocarditis, bioprosthetic endocarditis, mitral valve replacement, Valve in Valve, redo mitral valve replacement.
