# Supplementary figures and images for: Sapien ‘valve-in-valve’ implantation and valve leaflet resection for treating endocarditis of a bioprosthetic mitral valve: a case report
Source: Eur Heart J Case Rep. 2024 Feb 12;8(2):ytae078. doi: 10.1093/ehjcr/ytae078 (PMC10894001; doi:10.1093/ehjcr/ytae078)

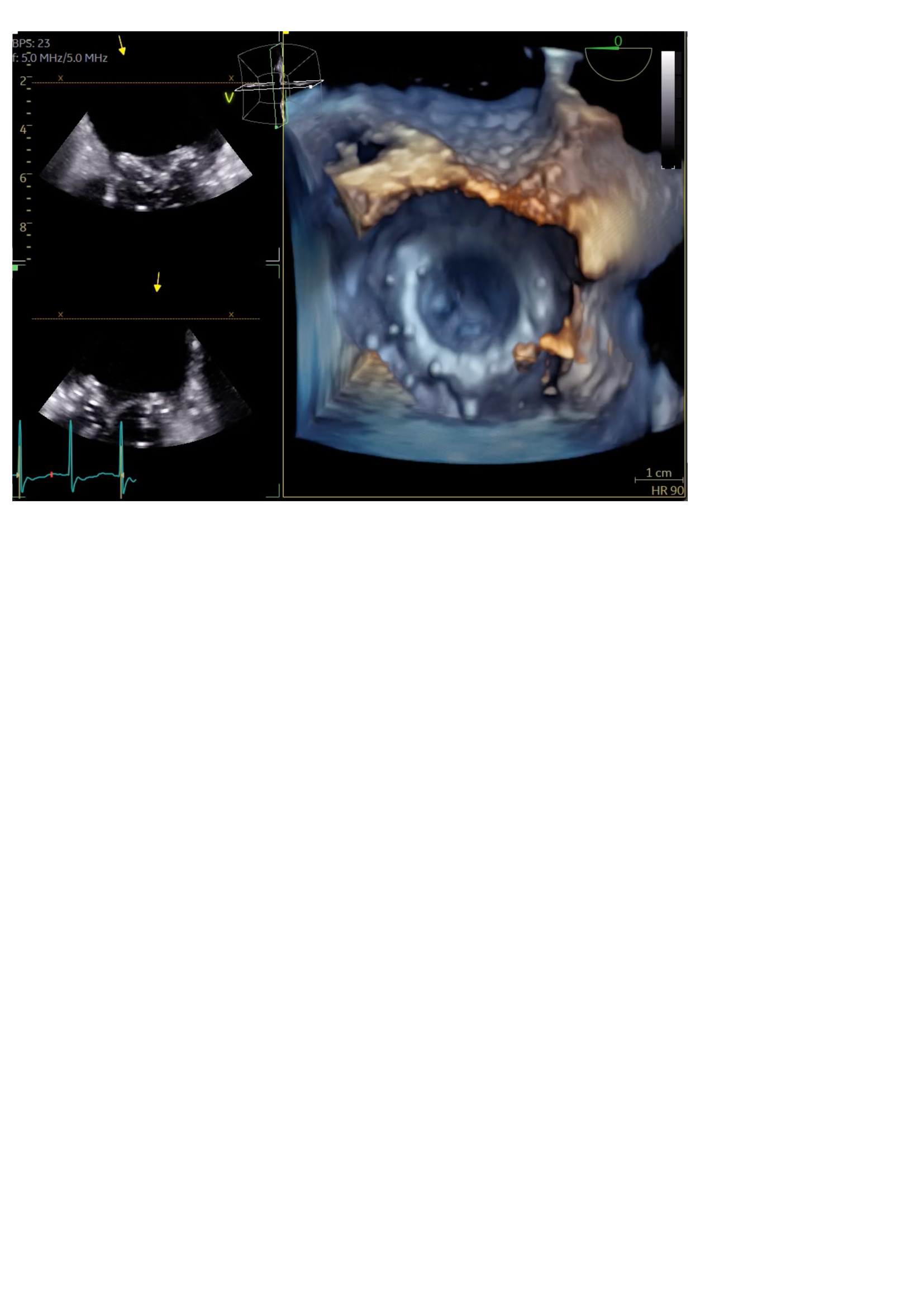

Supplement: ytae078_Supplementary_Data [file ytae078_supplementary_data.zip › figure3_still_image_video2_friedrich_pasic_falk.tiff]

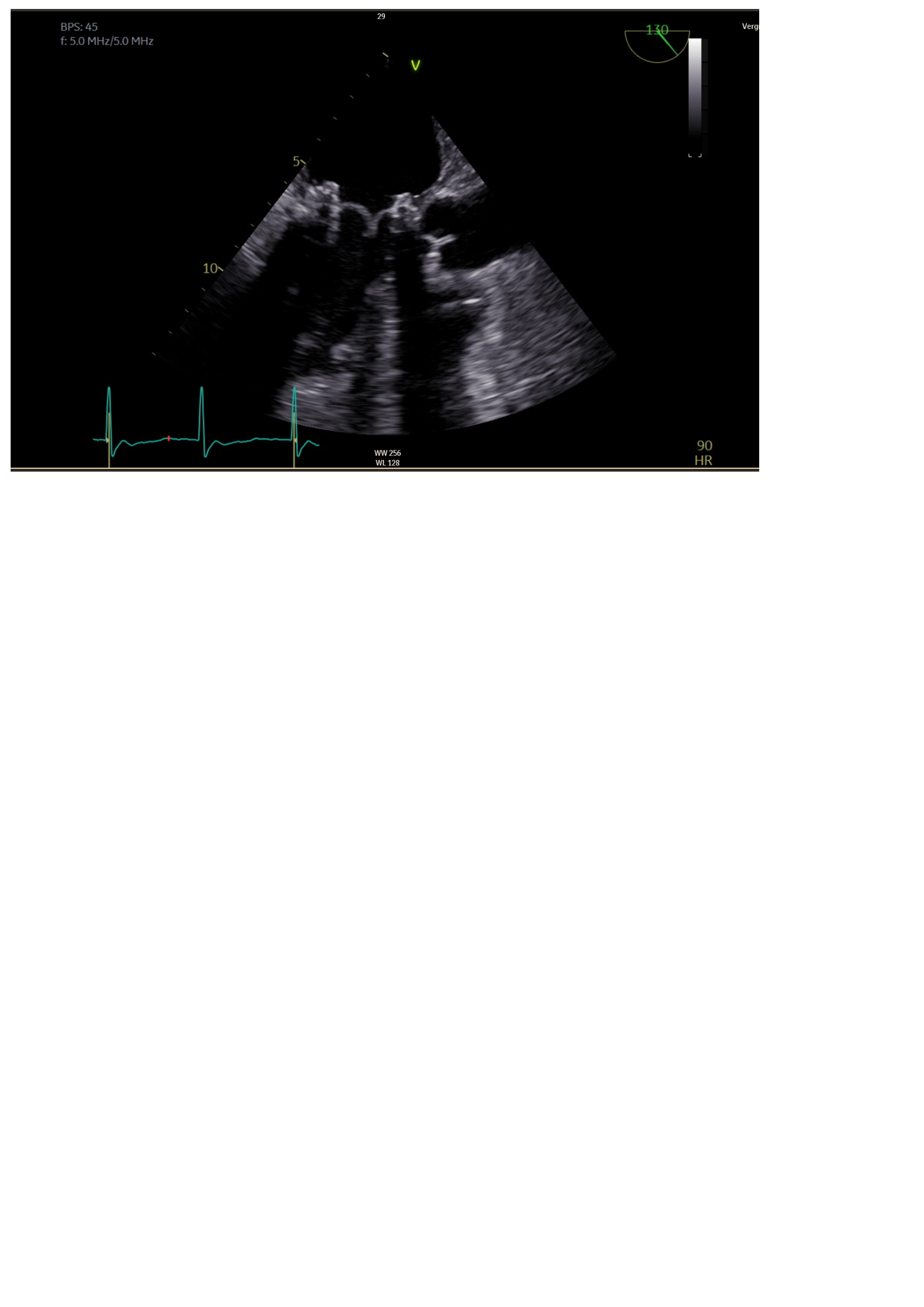

Supplement: ytae078_Supplementary_Data [file ytae078_supplementary_data.zip › figure4_still_image_video3_friedrich_pasic_falk.tiff]

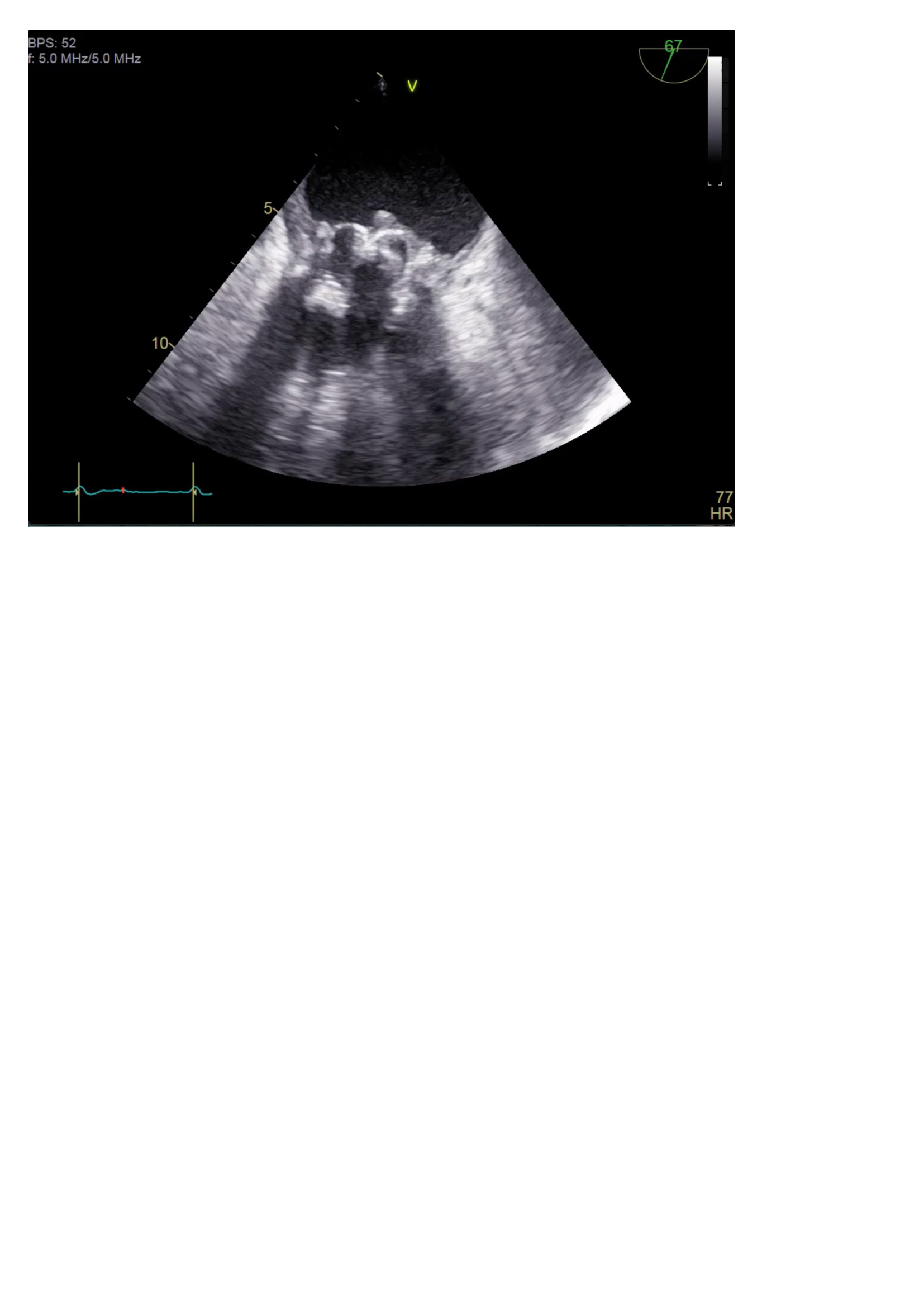

Supplement: ytae078_Supplementary_Data [file ytae078_supplementary_data.zip › figure2_still_image_viedo_1_friedrich_pasic_falk.tiff]
